# The protective role of rs56103835 against breast cancer onset in the Iranian population

**DOI:** 10.1002/mgg3.540

**Published:** 2019-01-31

**Authors:** Negin Naderi, Maryam Peymani, Kamran Ghaedi

**Affiliations:** ^1^ Department of Biology, Faculty of Basic Sciences, Shahrekord Branch Islamic Azad University Shahrekord Iran; ^2^ Department of Biology, Faculty of Sciences University of Isfahan Isfahan Iran

**Keywords:** breast cancer, miR‐323b, rs56103835 polymorphism

## Abstract

**Background:**

Breast cancer is one of the most common types of cancer among women and the highest cause of death due to cancer among women aged 40–45. SNPs can be used to identify disease‐related genes such as cancer as they can be genetic markers. Furthermore, SNPs in the molecular‐level miRNA structure are also associated with a set of cancers. Studies have shown that miR‐323b plays a tumor suppressor role by reducing the tissues and serum of the affected individuals. So far, no study regarding rs56103835 polymorphism in the precursor of miR‐323b has been conducted in the breast cancer. In this study, the association of this SNP with the incidence of breast cancer in the Iranian population has been investigated.

**Method:**

In order to correlate rs56103835 polymorphism with breast cancer, 161 patients and 162 healthy people as the control group were examined. They had been homogenized based on their age and gender. The genotype of individuals for the polymorphism was determined by the PCR‐RFLP method. The association of this polymorphism with the risk of breast cancer, the age of the onset of disease, and pathological characteristics of the patients was then analyzed.

**Results:**

The findings showed that there is no significant correlation between the frequency of its genotypes among the healthy and patient populations while the TT genotype increased the age of the disease in patients, as compared to other genotypes (*p* = 0.035, OR = 0.487).

**Discussion and Conclusion:**

The C allele is likely to inhibit the expression of *BRCA2* by interfering with the processing of this pre‐miRNA and increasing the expression of target genes such as *BRCA2.* Because one of the early onset genes in breast cancer is the *BRCA2*, the presence of any of C and T alleles can have a significant effect on the incidence of the disease. To further confirm this data, however, more molecular studies are needed.

## 1 INTRODUCTION

Breast cancer is ranked as the most prevalent disease among women. It causes 16% of deaths among women, as well as being the first cause of cancer death in the women aged 45–40 (Sadjadi et al., 2003). Unlike many other diseases, such as infectious diseases, that have a particular cause, most cancers are often the consequences of several factors including genetic/epigenetic features and lifestyle factors (Ambrosone, Hong, & Goodwin, 2015). Recently, researchers have been interested in the role of microRNAs (miRNAs) and their polymorphism in breast cancer (Bachour & Bennett, 2011; Pipan, Zorc, & Kunej, 2015).

It has been found that the abnormal expression of miRNAs may be related to the risk of cancer (Peng & Croce, 2016). Oncogenic miRNAs, commonly referred to as oncomirs, are usually deregulated in cancer cells whose targets are tumor suppressor genes such as miR‐21, miR‐96, and miR‐323 (Guttilla & White, 2009; Si et al., 2007). In contrast, tumor suppressor miRNAs (such as miR‐205, miR‐27b, and miR‐17–5p) are often expressed at lower levels in cancer cells, relative to the normal cells (Hossain, Kuo, & Saunders, 2006; Iorio, Ferracin, Liu, Veronese, & Spizzo, 2005; Tsuchiya, Nakajima, Takagi, Taniya, & Yokoi, 2006). Since these miRNAs act on genes that stimulate tumorigenicity (oncogenes), a decrease in their levels can increase the effects of oncogenes and the growth of cancer cells (Zhang, Pan, Cobb, & Anderson, 2007). Therefore, single nucleotide polymorphisms (SNPs) in the structure of these miRNA, called mirSNPs, may contribute to their deregulations, thereby contributing to the onset of cancer (Slaby, Bienertova‐Vasku, Svoboda, & Vyzula, 2012). mirSNPs may play an important role through their effect on the primary target gene transcription, the change of the pri‐miRNA/pre‐miRNA processing, or their interaction effects on miRNA‐mRNA (Ryan, Robles, & Harris, 2010). There are several data describing the association of rs1834306, rs2043556, rs3746444, rs4919510, rs2910164, rs11614913, rs895819, rs2292832, and rs6505162 with the risk of cancer (Hu et al., 2014). However, conclusions from the related studies have been inconsistent due to the heterogeneity of the type of cancer studied, the small size of the samples, and the different ethnicities of the patients. Therefore, there is the need to perform further research on the relation between cancer‐related mirSNPs and the risk of various types of cancer in each population. Although the identification of cancer‐related miRNAs has become increasingly popular on the basis of genetic link studies, research on the association of cancer‐related mirSNPs with the risk of various types of cancer has not yet been conducted (Hu et al., 2014).

Previous studies have shown that the expression of miR‐323b in serum and tumor tissue in breast cancer patients is reduced and this miRNA can play a suppressor role in the tumor genes of breast cancer patients (Zhu, Zheng, Wang, Sun, & Wang, 2014). Based on database prediction for miRNA targets, via Targetscan database, it has been found that the *BRCA2* gene is among the targets of miR‐323b. This gene is one of the early onset genes in breast cancer, with a remarkable expression effect on the early stages of breast cancer (Hedau et al., 2015; Narod, 2011).

Regarding the importance of polymorphisms in miRNAs, this study examined the protective role of rs56103835 polymorphism in the miR‐323 encoding gene and its association with the incidence of breast cancer. This miRNA is located on chromosome 14 and at the position q32.31. The rs56103835 polymorphism is located on the immature segment of miR‐323, which is considered as a nucleotide substitution of adenine with cytosine. The aim of this study was, therefore, to determine the relationship between this polymorphism and the incidence and progression of breast cancer in the population of Isfahan, Iran.

## 2 MATERIALS AND METHODS

### 2.1 Ethical Issues and the consent form

All participants, who had been informed, agreed to take part in this study by signing a consent form. All protocols were carried out based on the relevant guide lines for human sampling, and ethical issues were confirmed by the ethical committee of Islamic Azad University of Shahrekord.

### 2.2 Patients and controls

This case–control study was performed on 161 samples of breast cancer patients against 162 healthy women matched for age. Control samples with any history of cancer were excluded. Information about the population studied is shown in Table 1. The clinical and pathological characteristics of the patients were collected from the hospital, as summarized in Table 1. Blood samples were collected in EDTA‐containing tubes. Genomic DNA extraction was carried out from the collected blood samples according to the Genetbio (Korea).

**Table 1:** Patients’ characteristics

Variables	Numbers
Age, years	
≤45	61
>45	92
Grade	
I	19
II+III	56
Stage	
I+II	30
III+IV	41
Estrogen receptor	
Positive	72
Negative	47
Her2, *n*	
Positive	63
Negative	46
Progesterone receptor	
Positive	68
Negative	49

### 2.3 SNP genotyping and sequencing

Genotyping was performed on rs56103835 by the PCR‐RFLP method. To summarize, forward and reverse primers were 5'‐TCATCCTCAGGGTCCCATCCAC‐3 'and 5'‐ TGTCCCCTAAATCGGCATCAGG‐3', respectively, as designed by Oligo7. PCR mixture consisted of 7.5 μL of *Taq* DNA Polymerase Master Mix RED (Ampliqon), 0.6 μL of each primer, and 1.5 μL of genomic DNA and 4.8 μL of ddH2O at a final 15 μL. PCR products (221 bp) were then subjected to *NIa*III digestion at 37°C for 2 hr. In the presence of the C allele, there was no cut, whereas, in the presence of the T allele, the PCR product was cleaved to produce two fragments, 126 and 96 bp. To confirm the data obtained from genotyping, a number of samples from each of the homozygous and heterozygote genotypes were randomly assigned to sequencing through sending to Bioneer Co. (Korea). Sequencing results were analyzed by Chromas Lite software (version 2.0, Technelysium Pty. Ltd., Tewantin, Queensland, Australia).

### 2.4 Statistical analysis

Statistical analysis was performed using SPSS 26 software statistical package (IBM, Corp., Armonk, NY, USA). The categorical and continuous data were analyzed using chi‐square and *t* tests, respectively. The associations between genotypes and breast cancer were assessed by computing the odds ratio (OR) and 95% confidence intervals (CIs) from logistic regression analyses. *p* < 0.05 was considered as the significant difference between the samples.

### 2.5 Data source

miRNASNP database, version 2.0*,* was used to identify the polymorphisms related to miR‐323 and the selected rs56103835. We obtained the information about rs56103835, including its minor allele frequency and SNP 5'‐ and 3'‐flanking sequences, from the NCBI build 37.3 database, and got the SNP location from the 1,000 genome project. Using miRDB (http://www.mirdb.org/) and TargetScan (release 6.2, http://www.targetscan.org/) databases, we obtained the predictive targetome of rs56103835‐related miR‐323.

## 3 RESULTS

### 3.1 Optimization of PCR conditions with the designed primers and enzyme digestion to determine the genotype

**Figure 1:**
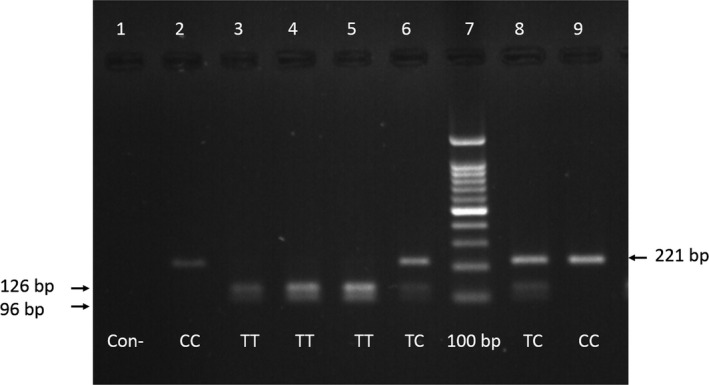
Optimization of PCR conditions and enzyme digestion to determine the genotype. The PCR product with 221 bp was then subjected to *NIa*III digestion at 37°C. In the presence of the C allele, products were 221 bp, and in the presence of the T allele, 126 bp and 96 bp were formed. Negative control (Con−) in this figure to show that lower bands are not primer dimer

**Figure 2:**
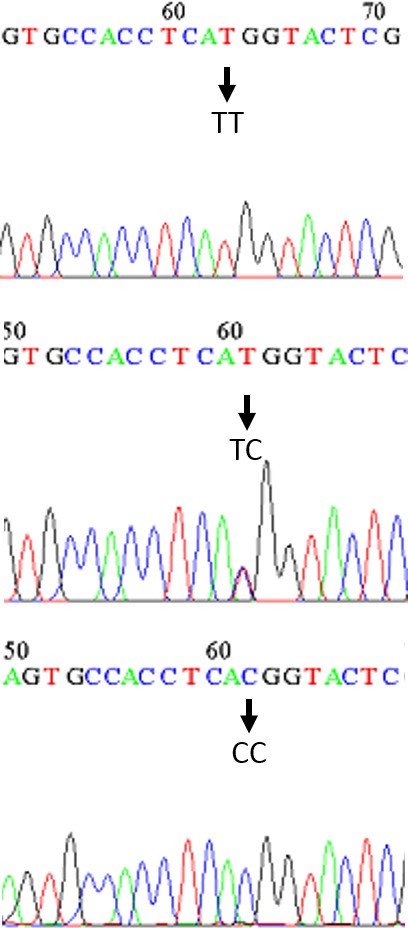
Sequencing chromatograms of rs4541843. T > C, and the sequencing chromatogram results of the genotypes TT, CT, and CC, respectively. Samples were chosen randomly

In the study of subjects, they were amplified using the PCR technique and specific region primers containing polymorphism rs56103835. For the propagation and formation of a specific bond without the presence of a non‐specific bond, the 63°C binding temperature was selected. To identify the rs56103835 genotype in each individual, PCR products were treated with the *NIa*III restriction enzyme. As shown in Figure 1, the fragments of 126 and 96 bp represented the genotype (TT), the fragments of 221, 126, and 96 bp stood for genotype (TC), and the fragments of 221 bp referred to the genotype (CC). The sequencing chromatogram results of the genotypes CC, TC, and TT for some of the samples confirmed the results of determining the genotype of the samples using the RFLP method (Figure 2).

### 3.2 Frequency of rs56103835 variation

The frequency of genotype and the allelic frequency of rs56103835 polymorphism in the population of the patients with breast cancer and healthy people can be seen in Table 2. Investigating the relationship between different genotypes of rs56103835 polymorphism and the risk of breast cancer showed that there was no significant difference between the genotypes in the two healthy and patient populations, and none of the genotypes was observed in this polymorphism, as compared to the TT genotype, which was considered as the reference genotype; so, there was no significant relationship with breast cancer.

**Table 2:** Association of the rs56103835 T > C polymorphism and risk of breast cancer

rs4541843	Case, *n* (%)	Control, *n* (%)	OR (95% CI)	*p*‐value
Codominant				
TT	90	101	1	–
TC	61	55	1.245	0.353
CC	10	6	1.870	0.243
Dominant				
TT	90	101	1	–
TC+CC	71	61	1.306	0.239
Recessive				
TT+TC	140	120	1	–
CC	25	41	1.722	0.304
Allele				
T	241	257	1	–
C	81	67	0.783	0.120

Note

CI, confidence interval; OR, odds ratio.

Given the dominance of allele C, the frequency of genotypes TC and CC in comparison to TT was calculated in the two healthy and patient populations, finding no significant correlation with the incidence of this disease at the level of <0.05. Given the dominance of the T allele, the frequency of total TC and TT genotypes in comparison to CC was calculated in the two healthy and patient populations and their association with breast cancer was studied. Interestingly, there was no significant correlation with the incidence of this disease at the level of <0.05. The findings, therefore, showed that this region could be a polymorphic region in the population and there was no significant correlation between the frequency of genotypes in the healthy and patient populations. Then, the association of the frequency of alleles with the risk of disease was investigated, showing no significant relationship with them (OR, 0.783; 95% CI, 0.576–1.066; *p* = 0.120). After that, the relation between different genotypes of polymorphism rs56103835 and the age of incidence of breast cancer was studied. According to Table 3, the results of this study showed that there was a significant correlation between the TC genotype of polymorphism rs56103835 and the age of the incidence of breast cancer in the patient population.This was in the sense that the genotypes TC significantly decreased the incidence of disease. On the other hand, the association of each of the alleles with the age of the disease was studied, showing that the presence of the C allele (OR, 0.635; *p* = 0.089) could decrease the incidence of the disease. These data, therefore, confirmed the effective association between allele C and the increased risk of breast cancer.

**Table 3:** Association of the *rs56103835* T > C polymorphism and age of onset in breast cancer

Age of onset (years)	≤45	>45	OR (95% CI)	*p*‐value
Genotype				
TT	26	55	1	–
TC	29	28	0.456	0.028
CC	4	6	0.709	0.617
CC/TC	33	34	0.487	0.035
Allele				
T	81	138	1	–
C	37	40	0.635	0.089

### 3.3 Correlation between rs56103835 T > C genotypes and the clinical characteristics of breast cancer patients

The relationship between different genotypes of *rs56103835 *polymorphism and histopathology features including ER +/−, PR +/−, and HER2 +/− was also studied in the patients with breast cancer; there was no significant correlation between the frequency of genotypes and the presence or absence of these factors in the infected people. These results have been summarized in Tables 3 and 4.

**Table 4:** Correlation between *rs56103835* T > C genotypes and clinical characteristics of breast cancer patients

	*rs56103835* T > C genotype			
Variables	TT TC CC			*p*‐value
Age, years				
≤45	26	29	4	0.085
>45	55	28	6	
Grade, n				
I	10	8	1	0.543
II+III	37	16	3	
Stage, n				
I+II	13	14	3	0.398
III+IV	24	15	2	
Estrogen receptor, n				
Positive	42	24	6	0.586
Negative	24	20	3	
Her2, n				
Positive	36	25	3	0.468
Negative	26	16	5	
Progesterone receptor, n				
Positive	37	27	4	0.730
Negative	28	16	4	

Note

HER2, human epidermal growth factor receptor 2.

### 3.4 Suggesting the possible roles of has‐miR‑323b in cancer signaling pathways with the Molecular signaling pathway enrichment analysis of has‐miR‑323b targetome

In order to understand how miR‐323b could be related to cancer, a molecular signaling pathway enrichment analysis was conducted. Moreover, the predicted targetome of miR‐323b with TargetScan database was found and used so as to conduct more molecular enrichment analysis. When official gene symbols of miR‐323b targetome were put into a functional annotation tool of DAVID database, ia statistically significant relation with KEGG signaling pathways was found; the most significant ones were in cancer pathways were such as TGF‐beta, ErbB, Cell cycle, p53, and Jak‐STAT signaling pathways (https://david-d.ncifcrf.gov/) (Table 5).

**Table 5:** The molecular pathway enrichment analysis of has‐miR‐323b

	Kegg pathway	Number of genes in the pathway	*p*‐value
1	Pathway in cancer	68	6.3‐E4
2	Melanoma	21	1.3E−3
3	Focal adhesion	45	1.3E−3
4	T‐cell receptor signaling pathway	28	1.5E−3
5	Insulin signaling pathway	32	3.1E−3
6	Adherens junction	21	3.8E−3
7	Type II diabetes mellitus	15	3.9E−3
8	Colorectal cancer	22	4.9E−3
9	Neurotrophin signaling pathway	29	6.1E−3
10	Chronic myeloid leukemia	20	6.3E−3
11	Renal cell carcinoma	19	6.5E−3
12	Ubiquitin mediated proteolysis	31	7.3E−3
13	TGF‐beta signaling pathway	22	7.6E−3
14	Adipocytokine signaling pathway	18	9.3E−3
15	ErbB signaling pathway	21	1.5E−2
`16	Endocytosis	36	3.4E−2
17	MAPK signaling pathway	49	3.7E−2
18	Pancreatic cancer	17	3.8E−2
`19	Cell cycle	26	3.9E−2
20	Small cell lung cancer	19	4.0E−2
21	RNA degradation	14	4.8E−2
22	Glioma	15	5.0E−2
23	Aldosterone‐regulated sodium reabsorption	11	5.2E−2
24	Acute myeloid leukemia	14	5.5E−2
25	Amyotrophic lateral sclerosis (ALS)	13	5.9E−2
26	Jak‐STAT signaling pathway	30	6.0E−2
27	Prostate cancer	19	6.6E−2
28	Cell‐adhesion molecules (CAMs)	26	6.8E−2
29	Biosynthesis of unsaturated fatty acids	7	7.7E−2
30	Valine,leucine, and isoleucine degradation	11	7.9E−2
31	Long‐term potentiation	15	8.6E−2

## 4 DISCUSSION AND CONCLUSION

In this study, first, the association of rs56103835 polymorphisms in the pre‐miR‐323b sequence with the risk of breast cancer was examined in a population of breast cancer in Isfahan, Iran. The findings showed that this region was a polymorphic one in the population and there was no significant correlation between the frequency of genotypes in the healthy and patient populations. It seemed that the effect of a miRNA‐related SNP depended on its intrinsic effect on its miRNA function as well as the role of miRNA in controlling the key genes involved in cancer. The desired polymorphism was a single‐nucleotide transformation T > C, such that the C allele was based on the dbSNP database as a risk allele and with a frequency of 30%. In this study, the frequency of this allele in both healthy and patient populations was 20% and 25%, respectively, the frequency of allele C in the total population was 22%, and there was no significant difference in the allele frequency between the control and patient populations. Also, there was no significant difference between the frequencies of genotypes in these two populations and each of the observed genotypes of this polymorphism, as compared to the TT genotype as the reference genotype in this population, showing no relation with a significant risk of breast cancer at the level of <0.05. The aim of this study was to investigate the relationship between different pathological parameters of the disease, including the presence or absence of estrogen receptor, progesterone, HER2 and stage disease, grade disease, metastasis, and mortality rate, with polymorphism rs56103835. There was no significant relationship between each of these parameters and the frequency of genotypes in the population of patients whose details of the parameters were available. Finally, the relationship between the age of the disease in the patients and the frequency of each of the genotypes was investigated, showing that the CT genotype of polymorphism rs56103835 with the age of the incidence of breast cancer was significantly correlated with the patient population (*p* = 0.028, OR = 0.0456). Considering the dominance of C allele, the frequency of the total CC and CT genotypes, in comparison to TT, in the patients was calculated based on the age of the patients’ incidence, and their association with the age of breast cancer was studied. The results showed that the TT genotype, in comparison to other genotypes, increased the age of the disease (*p* = 0.035, OR = 0.487). Then, the association of each allele with the age of the disease was also studied, showing that the presence of C alleles with *p* value = 0.08 could possibly provide a basis for reducing the incidence of disease.

In this regard, Zhu and colleagues conducted research on the changes upon the expression of miR‐323b in the patients with breast cancer. They found that the expression of miR‐323b in serum and tumor tissue in breast cancer patients was reduced, showing that miRNA played a suppressive role in the tumor genes of breast cancer patients (Zhu et al., 2014). Based on the predictive databases for miRNA targets, including the Targetscan base, *BRCA*2 gene was identified as one of the miR‐182 targets. This gene could be considered as one of the early onset genes in breast cancer, with a significant expression in the early stages of breast cancer (Narod, 2011). Increasing the expression of this gene led to cancer progression (Montazeri, Sadighi, Farzadi, Maftoon, & Vahdaninia, 2008). By reducing the expression of miR‐323b, the targets of this miRNA could increase the expression in the target tissue, which can be referred to as *BRCA2*, thereby raising the expression of the gene, the age of the disease, and the progression of the disease. According to the results, the association of rs56103835 polymorphism with the incidence and progression of breast cancer for the first time was investigated; given that the desired polymorphism in the pre‐miR‐323b structure was predicted, the C allele probably inhibited *BRCA2* expression by interfering with the processing of this pre‐miRNA, causing an increase in the expression of target genes such as *BRCA2*. Since one of the early onset genes in *BRCA2* gene is breast cancer, the presence of any of the C and T alleles can have a significant effect on the incidence of disease. However, confirmation of this function of polymorphism requires more studies.

## CONFLICT OF INTEREST

None declared.
